# The different faces of GATA2 deficiency: implications for therapy and surveillance

**DOI:** 10.3389/fonc.2024.1423856

**Published:** 2024-06-27

**Authors:** Luca Vinci, Brigitte Strahm, Carsten Speckmann, Miriam Erlacher

**Affiliations:** ^1^ Department of Pediatrics and Adolescent Medicine, Division of Pediatric Hematology and Oncology, Medical Center, Faculty of Medicine, University of Freiburg, Freiburg, Germany; ^2^ Institute for Immunodeficiency, Center for Chronic Immunodeficiency (CCI), Medical Center, Faculty of Medicine, University of Freiburg, Freiburg, Germany; ^3^ Department of Pediatrics and Adolescent Medicine, University Medical Center Ulm, Ulm, Germany

**Keywords:** GATA2, HSCT, mds, myeloid neoplasia, Cancer predisposition

## Abstract

GATA2 deficiency is one of the most common genetic predispositions to pediatric myelodysplastic syndrome (MDS) in children and adolescents. The wide spectrum of disease comprises, among others, hematological, immunological and pulmonary manifestations, as well as occasionally distinct organ anomalies. Due to the elevated risk of progression, nearly all individuals with GATA2-related MDS eventually undergo a hematopoietic stem cell transplantation (HSCT) at some point in their lives. Nevertheless, the optimal timing, method, and even the indication for HSCT in certain cases are still matter of debate and warrant further research. In this article, we report five patients with different hematological and immunological manifestations of GATA2 deficiency ranging from immunodeficiency and refractory cytopenia of childhood without chromosomal aberrations to relapsed MDS-related acute myeloid leukemia. We discuss the adopted strategies, including intensity of surveillance, indication and timing of HSCT, based on morphological, clinical and molecular markers, as well as individual patient needs. We conclude that a better characterization of the natural disease course, a better understanding of the prognostic significance of somatic aberrations and a thorough evaluation of patients´ perspectives and preferences are required to achieve a personalized approach aimed at improving the care of these patients.

## Introduction

A significant portion of pediatric malignancies arises from germline mutations in cancer predisposition genes (1). The latest International Consensus Classification (ICC) categorizes hematologic neoplasms with germline predisposition based on the presence of a constitutional disorder and the involvement of multiple organ systems (2, 3). These include predisposition due to germline mutation in *GATA2*, *SAMD9* or *SAMD9L* genes as well as classical bone marrow failure (BMF) syndromes such as Fanconi anemia, Shwachman-Diamond syndrome, and telomere biology disorders.

The potential strategies for the management of patients with genetic predisposition to myeloid malignancy span from vigilant monitoring without intervention to the administration of pharmacological therapy and hematopoietic stem cell transplantation (HSCT). Each of these approaches is associated with specific risks and benefits and, due to the rarity of these conditions, there is a lack of sufficient data for evidence-based decision-making. Furthermore, the multisystemic manifestation often constitutes a major challenge to the treating physician.

GATA2 deficiency is one of the most common genetic predispositions to myelodysplastic syndrome (MDS), accounting for 7% of all pediatric patients diagnosed with MDS initially thought to be *de novo* (4). The initial manifestation of MDS in these patients includes all stages of disease, ranging from refractory cytopenia of childhood (RCC), to MDS with excess blasts (MDS-EB) or MDS-related acute myeloid leukemia (MDR-AML) (2). In addition to its hematologic manifestations, the spectrum of GATA2-related disease encompasses, among others, immunodeficiency with monocytopenia, B- and NK-cell lymphopenia, pulmonary disease, typically in the form of pulmonary alveolar proteinosis (PAP), as well as syndromic features like congenital deafness and lymphedema (5). The outcome of HSCT in pediatric patients with GATA2-related MDS depends on blast count and cytogenetics. Patients with RCC and normal karyotype have an excellent outcome, while an increased blast count and the presence of monosomy 7 are associated with poorer prognosis (6). Due to the high risk of progression to advanced disease observed in this population, careful consideration of the optimal timing and method of treatment is essential. Moreover, a standardized approach for the management of patients primarily affected by the non-hematological manifestations of GATA2 deficiency still has to be identified.

In pediatric MDS, the reduction of disease burden through the application of intensive chemotherapy before the allograft may be beneficial for patients with more advanced disease [for instance for those patients with > 20% blasts in bone marrow (BM)] (7, 8). However, due to the rarity of the condition and the lack of studies testing cytoreductive therapy prior to HSCT in a prospective and systematic manner - potentially stratifying for the stage and type of MDS (primary, treatment-related, or associated with a germline predisposition) - a standardized approach for managing MDS-EB before HSCT is currently lacking (9).

In this report, we describe the clinical course and treatment strategies employed in five patients with GATA2 deficiency, illustrating the challenges faced in their management and the need for personalized and shared decision-making in their clinical care.

## CASE 1

A 12-year-old girl was referred to the hospital because of productive cough, paleness and recently appeared edemas of hands and feet. The blood tests showed an isolated normocytic anemia (Hb 5.4 g/dL, requiring transfusion) and elevated inflammatory markers (CRP 179 mg/L). Imaging by ultrasound and magnetic resonance revealed mild pericardial effusion, enlarged axillary lymph nodes and bilateral arthritis of the elbows. Six months before, the girl’s mother had passed away because of a septic pneumonia in the setting of PAP related to GATA2 deficiency. The genetic condition in the mother was characterized, along with its pulmonary manifestation, by profound B- and NK-cell lymphopenia with recurrent pneumonias and a human papillomavirus (HPV) driven neoplasia of the genital tract.

In view of the positive family history, a genetic testing was performed in peripheral blood (PB) and the suspected *GATA2* mutation [NM_032638.4, c.351C>G; (p.T117T)] was detected. Analysis of the lymphocyte subsets showed a normal distribution of T-cells, but profound B-and NK-cell lymphocytopenia as well as absence of myeloid and plasmacytoid dendritic cells. An extensive infectious work up did not reveal any infection as underlying cause for the inflammation and a systemic steroid therapy was initiated. The arthritis and pericardial effusion improved; however, the patient required a second transfusion of packed red blood cells three months after the first. At this point, BM aspirate and trephine biopsy were performed and showed a normocellular marrow with moderate dysplasia, resulting in the diagnosis of normocellular RCC. Metaphase cytogenetics revealed monosomy 7. These findings were confirmed in the follow-up assessments conducted four months later. The patient had no matched family donor, but a 9/10 HLA matched unrelated donor (UD) was identified. HSCT was scheduled, but had to be postponed because of a pneumocystis jirovecii pneumonia that was successfully treated with cotrimoxazole and steroids. The girl then successfully underwent HSCT with a BM graft after a reduced toxicity conditioning regimen with thiotepa (TT), treosulfan (Treo) and fludarabine (Flu), serotherapy with anti-T-lymphocyte globulin (ATG) and graft versus host disease (GvHD) prophylaxis with cyclosporine A (CSA) and short-course methotrexate (MTX). She engrafted with full donor chimerism and developed acute GVHD grade II of the skin, which resolved with topical and systemic steroids. Seven years after HSCT, the patient is in excellent clinical condition with normal blood counts, full immune recovery and no history of infectious complications.

### Considerations

This case illustrates characteristic features of GATA2 deficiency such as immunological manifestations (immunodeficiency/immune dysregulation) and MDS with monosomy 7 (5).

The known family history facilitated a prompt diagnosis of GATA2 deficiency in this patient. GATA2 deficiency is characterized by an autosomal dominant inheritance. However, with the majority of mutations occurring *de novo*, a positive family history is present in only 20 - 30% of the cases (4, 10). The *GATA2* variant identified in this family is a so-called “synonymous mutation”. The mutation does not alter the amino acid sequence of the encoded protein but leads to an aberrant mRNA splicing that introduces a new stop codon (11). Silent *GATA2* mutations might also result in nonsense-mediated mRNA decay and account for 8% of cases with GATA2 deficiency in a recent screening of the EWOG-MDS cohort (12). Remarkably, the identical mutation resulted in distinct phenotypic presentations between mother and daughter, emphasizing the considerable variable expressivity of GATA2 deficiency, even within a single family.

Immune-related phenotypes are also frequent and may be both related to immunodeficiency and immune dysregulation. The most frequent infectious complications in GATA2 deficiency are HPV, fungal and disseminated mycobacterial infections. On the contrary, the occurrence of pneumocystis jiroveci pneumonia has been rare, highlighting the considerable heterogeneity of the immunodeficiency spectrum in GATA2 deficiency (13). B-cell lymphopenia with loss of progenitors in PB and BM is instead typical of pediatric GATA2 deficiency (14, 15), where studies of lymphocyte subpopulations often reveal preserved T-lymphocytes with severe deficiency of monocytes and B- and NK-cells. Humoral immunodeficiency may not manifest at diagnosis and then develop subsequently (5, 16). Autoimmune manifestations, including arthritis and serositis as in our case, have been extensively reported, whereas hyperinflammatory states appear to be less frequent (17). In our case, the autoimmune manifestations might also be associated with the MDS diagnosed during further work-up (18).

Monosomy 7 is the most frequent cytogenetic abnormality in GATA2-related MDS, occurring in approximately two thirds of the patients. Like in other MDS cases, the presence of an abnormal karyotype with monosomy 7 is associated with rapid progression to advanced disease and a worse disease-free-survival (DFS) after HSCT (4, 6). The presence of monosomy 7 therefore clearly indicated the need for HSCT in this patient (19).

The European Working Group of MDS in Childhood (EWOG-MDS) originally recommended a myeloablative conditioning regimen consisting of busulfan, cyclophosphamide and melphalan (BuCyMel) in all MDS patients, including RCC cases. However, the very low risk of relapse in the presence of a rather high transplant-related mortality for patients with RCC later prompted the adoption of less toxic regimens (20). As a result, the current recommendations from the EWOG-MDS suggest a conditioning regimen with TTTreoFlu for RCC with monosomy 7 and normo- or hypercellular RCC with normal karyotype. In contrast, patients with hypocellular RCC and normal karyotype currently receive a conditioning regimen consisting of Treo and Flu (21).

#### Learning points:

- GATA2 deficiency can be caused by silent mutations, mandating their inclusion into standard diagnostic tools for individuals with GATA2 deficiency phenotype.
- B- and NK-cell lymphopenia, with or without hypogammaglobulinemia, is common in GATA2 deficiency and evaluation of lymphocyte subset distribution in PB is a helpful diagnostic tool.
- It is essential to perform thorough BM studies including trephine biopsy and cytogenetic analysis even in patients with predominant immunologic rather than hematologic manifestations of GATA2 deficiency.

## CASE 2

A 16-year-old girl was referred to the hospital due to massive hepatosplenomegaly and skin rashes. These presented as hives on her thighs and resembled erythema nodosum on her upper arms. The past medical history was unremarkable with the exception of migraine with aura. Her family history was empty with regard to hematological diseases. Her complete blood count showed leukocytosis (WBC 24.3 x 10^9^/L), moderate thrombocytopenia (Plt 69 x 10^9^/L) and normocytic anemia (Hb 9.1 g/dL). Analysis of the PB smear revealed the presence of circulating myeloid and erythroid precursors with 1% myeloblasts. The BM aspirate showed increased cellularity and pronounced dysplastic changes in all lineages with micromegakaryocytes, left-shifted myelopoesis and a macrocytic maturation of the erythropoiesis. Myeloblasts were not increased (3%), whereas an increase in partially immature monocytic cells was seen (10–20%). Cytogenetic analysis identified monosomy 7. The genetic analysis from hair bulbs identified an intronic germline mutation in *GATA2* (c.229 + 1dupG); in addition, somatic pathogenic mutations in *WT1*, *SETBP1* and *ASXL1* were detected in PB. A re-assessment performed after 4 weeks confirmed the morphological, genetic and laboratory findings resulting in the diagnosis of an overlap syndrome between myeloproliferative neoplasm (MPN) and MDS (MDS/MPN). In the absence of a HLA compatible donor the girl underwent haploidentical HSCT from her father, who had tested negative for the *GATA2* mutation during the genetic testing that had been offered to the family after the diagnosis of the patient. In the absence of an excess of blasts, the patient did not receive any induction chemotherapy and underwent upfront HSCT. She received a TCRαβ/CD19-depleted peripheral blood stem cell (PBSC) graft after a conditioning regimen with BuCyMel and serotherapy with ATG. She had a slightly delayed hematologic recovery with full donor hematopoiesis and is now in good clinical condition 6 months after HSCT.

### Considerations

Although the two first cases (Case 1 and 2) share clinical presentations with autoimmune/inflammatory manifestation, their hematological presentations differ significantly. Case 1 presented with isolated anemia, whereas Case 2 demonstrated leukocytosis, hepatosplenomegaly and a hypercellular BM indicating a strong myeloproliferative component. The current ICC defines MDS/MPN as the presence of cytopenia without excess blasts, concomitant leukocytosis (≥ 13 x 10^9^/L) or thrombocytosis (≥ 450 x 10^9^/L), evidence of clonality as well as exclusion of BCR-ABL1 translocation, genetic abnormalities typical of myeloid/lymphoid neoplasms with eosinophilia, tyrosine kinase gene fusions or t(3;3)(q21.3;q26.2), inv(3)(q21.3q26.2), or del(5q) (3). The cause of the myeloproliferative presentation in this patient remains uncertain and might be the result of acquired mutations in a specific cell of origin.

Based on the diagnosis of MDS/MPN, monosomy 7 and the presence of several somatic mutations indicating high risk disease in the setting of AML, we expected a rather high risk of relapse in this case. An intensive conditioning regimen consisting of BuCyMel, as recommend for MDS-EB and MDR-AML in the current EWOG-MDS guidelines, was therefore applied (21).

When choosing a family donor for HSCT, the exclusion of *GATA2* germline mutations in the donor is of utmost importance, even in the absence of clinical suspicion. Failure to recognize familial disease increases the risk for fatal disease due to impaired immune reconstitution after HSCT and donor-derived post-HSCT MDS/MDR-AML (22). Being aware of the risk of unrecognized germline predisposition syndromes in pediatric patients with MDS, the EWOG-MDS group recommends that, even in the absence of known germline variants, BM analysis including trephine biopsy and cytogenetics should be performed in all potential family donors to exclude unrecognized familial disease.

#### Learning points:

• In MDS without excess blasts, other diagnostic features such as the diagnosis of MDS/MPN, cytogenetic aberrations or somatic mutations indicate high risk disease and might warrant an intensive conditioning regimen.
• Genetic testing of potential family donors is mandatory for patients with a known germline genetic variant predisposing to a myeloid neoplasm.

## CASE 3

A 10-year-old boy presented with a febrile episode and his blood count showed thrombocytopenia (Plt 69 x 10^9^/L), leukocytopenia (WBC 1,7 x 10^9^/L) with moderate neutropenia (ANC 0,5 – 1 x 10^9^/L) and monocytopenia (AMC 0,2 – 0,5 x 10^9^/L). An initial BM aspirate resulted in the exclusion of acute leukemia. Immunological testing revealed reduced levels of IgG and IgA with normal response to vaccine stimuli. Lymphocyte analysis indicated decreased levels of B- and NK-cells. Testing for the causes of BMF known at that time yielded negative results. The clinical course remained uneventful and the patient was lost to follow-up until he presented again at the age of 17 years complaining of palmar and plantar warts. A large hydrocele testis was diagnosed and wound healing after surgery was prolonged. With the exception of an isolated mild thrombocytopenia (Plt 104 x 10^9^/L) the blood count had normalized. Based on the past medical history and the clinical presentation, GATA2 deficiency was suspected and a heterozygous mutation in *GATA2* was identified [c.1109G>T; (p.C370F)]. BM biopsy showed dysplastic megakaryopoiesis, hypoplastic erythropoiesis and left-shifted myelopoiesis and was compatible with normocellular RCC. Cytogenetic testing revealed trisomy 8, while the NGS panel analysis for genes associated with myeloid disease yielded negative results. In the absence of high risk features, a watchful waiting with complete blood counts (CBCs) every three months and BM diagnostics once yearly was chosen, while a donor search was initiated. In addition, the patient received subcutaneous immunoglobulin substitution indicated by critically low IgG levels. In the further clinical course the patient suffered from recurrent hydrocele testis. The blood counts revealed undulating values of white blood cells and platelets, however never associated with severe neutropenia, nor with the need for transfusions or with bleeding diathesis. The BM findings, including the trisomy 8, remained stable, without detection of somatic mutations. The patient was 22 years old at the last visit and in very good clinical condition.

### Considerations

In this case, GATA2 deficiency was suspected 7 years after the initial presentation with pancytopenia based on the typical extra-hematological manifestations with primary lymphedema of the genitals as well as palmar and plantar warts (23). Subsequent reevaluation of the first trephine biopsy performed at the age of 10 revealed a slightly hypocellular BM with mild dysplasia and significant reduction of B-cells, which is a typical feature of GATA2 deficiency (15).

GATA2 deficiency can manifest with diverse immunological features, linking it to various clinical entities once believed to be separate and distinct; these include MonoMAC syndrome (monocytopenia with Mycobacterium avium complex infection), DCML deficiency (dendritic cell, monocyte, B-, and NK-cell deficiency) and Emberger syndrome (MDS with lymphedema) (24). The patient described in this case fulfills characteristics of both DCML deficiency and Emberger syndrome. Lymphedema, in particular, is notably present in 11–20% in GATA2 patients; it typically affects the lower limbs and frequently involves the genitals in the form of a hydrocele (5, 14, 25). Saettini et al. have recently challenged the previous notion that lymphedema is never observed in patients with missense mutations, which was considered the sole genotype-phenotype correlation observed in GATA2 deficiency. Their findings reveal that lymphedema can indeed also occur in patients with missense mutations, as also confirmed by our case (26). Though heterogeneous, the B and NK-cell lymphopenia observed in this patient is a frequently encountered immunophenotypic hallmark of the disease (16). Notably, unlike case 1, clinically significant hypogammaglobulinemia was already evident at the time of diagnosis in this patient.

Isolated trisomy 8 is the second most common cytogenetic abnormality in GATA2 deficiency, with an estimated average rate of 15% (23), and was included in the intermediate cytogenetic risk group in the revised International Prognostic Scoring System (IPSS-R) for MDS (27). Patients with GATA2 deficiency and trisomy 8 may present in all stages of disease, ranging from RCC to MDS-EB and MDR-AML (4, 26). The first BM analysis performed in our case unfortunately did not include cytogenetic studies. However, since first detection of trisomy 8 the patient showed a stable disease course over more than 4 years. Calvo and Hickstein recently reported a case of a GATA2-related MDS in an adult woman with a documented 26-year history of trisomy 8 (16). The prognostic significance of trisomy 8 in the setting of GATA2-related MDS is thus still to be elucidated, and based on the currently available data it does not constitute an immediate indication to HSCT.

While the indication for HSCT is straightforward in patients with GATA2 deficiency and MDS exhibiting features such as an excess blasts, cytogenetic abnormalities indicating a high risk of progression, such as monosomy 7, or cytopenias accompanied by severe neutropenia and/or transfusion dependency for red blood cells and/or platelets, the decision whether to proceed with HSCT becomes more challenging in the absence of such features. Additional indications include clinically relevant immune deficiency or recurrent infections. The patient described in this case is diagnosed with immune deficiency including B-cell lymphopenia and hypogammaglobulinemia. However, he has been successfully treated with immunoglobulin replacement, and the recurrent warts have no impact on his quality of life. In this setting, the possibility of a preemptive HSCT was discussed with the patient and the family, along with the alternative of a thorough watchful waiting under the availability of multiple potential donors in case HSCT becomes necessary. The latter emerged to be the most fitting solution for our patient’s needs and proved to be safe over the years.

#### Learning points:

- In contrast to monosomy 7, the presence of trisomy 8 in GATA2-related MDS is not clearly associated with disease progression and does not constitute a clear indication to HSCT.
- In adolescents and young adults the possibility of preemptive HSCT needs to be thoroughly discussed.
- Diagnosis of GATA2 deficiency remains challenging because of its often incomplete phenotype at time of presentation; the disease should be suspected in patients presenting with dermatologic manifestations such as recurrent lymphedema or history of recurrent opportunistic infections without evidence of other primary immune deficiencies.

## CASE 4

A 23-year-old woman was referred to the hospital to perform further diagnostics because of a clinical history of lymphedema of her right leg, susceptibility to viral infections, active HPV infection and severe monocytopenia (AMC 0.05 x 10^9^/L) with neutropenia (ANC 0.8 x 10^9^/L). The complete blood count revealed a macrocytic anemia (Hb 10.2 g/dL, MCV 105 fL) and leukocytes at the lower limits of normal (WBC 3.1 x 10^9^/L). The biochemical profile showed normal levels of IgG. Among other tests, a genetic panel for inherited BMF was performed and a pathogenic germline mutation in *GATA2* [c.1192 C>T; (p.R398W)] was identified. Although not genetically tested, the family history of the patient´s mother was also suggestive of GATA2 deficiency: she had a long history of lymphedema of both legs following gynecological surgery, had recurrent mycobacterial infections and died at the age of 58 because of a bacterial pneumonia. The patient´s BM was hypocellular, with reduced myelopoiesis and megakaryopoiesis and showed no excess blasts. Erythropoiesis was reduced and patchily distributed, leading to a morphological diagnosis of refractory cytopenia with multilineage dysplasia, which is the adult equivalent of hypocellular RCC in childhood. Cytogenetic analysis per FISH excluded a monosomy 7, deletion in 7q or trisomy 8. NGS panel analysis revealed a secondary mutation in *ASXL1* at a VAF of 5%. The patient was informed about her disease and the risks associated with progression of MDS, received genetic counselling and clearly indicated the intention to undergo a HSCT. HLA typing was performed and a donor search was initiated. Due to the fertility concerns related to HSCT, the patient expressed a desire to defer the procedure, allowing for the possibility of childbirth before undergoing HSCT. Because of the clonal evolution with somatic *ASXL1* variant, we planned an intensive surveillance with regular complete blood counts and a BM examination with trephine biopsy, cytogenetics and NGS panel analysis every 6 months. The indication for HSCT will be periodically re-evaluated based on morphologic, cytogenetic and molecular findings, as well as the patient’s preferences.

### Considerations

The manifestation with lymphedema, immunodeficiency with monocytopenia, susceptibility to viral infections and active HPV-infection constitute the hallmarks of GATA2 deficiency in this patient. The family history of lymphedema and increased susceptibility to infections should have triggered a stronger suspicion of a genetic disorder, potentially resulting in an earlier diagnosis.

In the absence of transfusion dependency, severe neutropenia or high-risk cytogenetic abnormalities, the patient has no clear indication to HSCT. However, the lifelong prevalence of myeloid neoplasia in individuals with GATA2 deficiency is estimated to be approximately 75%, with the risk increasing rapidly as age advances (14, 23).

In an effort to better specify the individual risk of progression for the patient, an NGS analysis was conducted, revealing a relatively small clone in *ASXL1*. Recent analyses showed that *ASXL1* mutations are common in the premalignant state of GATA2 deficiency and that patients harboring these alterations have lower probabilities of survival (28, 29). Interestingly, *ASXL1* mutations were found far more frequently in female than male patients and, although discording data is present in literature, they do not appear to significantly correlate with abnormal cytogenetics. Deciding whether the existence of an isolated *ASXL1* clone, along with its size, is enough to warrant HSCT in the absence of cytogenetic abnormalities or other clear indications cannot be conclusively determined based on the currently available data. So far, no somatic gene mutation in any gene has been established as an independent factor able to lead decision-making in GATA2-related MDS.

In such circumstances, it is crucial to provide patients and their caregivers with thorough education about the disease and its possible courses. Subsequent discussions should delve into the potential risks and benefits of HSCT. In our specific case, the patient was informed about the elevated risk of progression in GATA2-related MDS or AML, the less favorable prognosis of HSCT in advanced disease compared to RCC, and the current lack of a clear indication for HSCT, given the uncertainty surrounding *ASXL1* as an indicator of evolution. It was also explained to the patient that, while the outcomes of HSCT for GATA2-related RCC have been demonstrated to be excellent, the procedure still carries potentially life threatening risks of GVHD and graft failure. Furthermore, infertility and the sexual health issues related with chemotherapy and HSCT were thoroughly discussed and considered in the decision-making process.

Given the high risk of progression of the hematologic disease of the patient, but also considering the uncertainty related to the rate with which progress could take place and taking into account her expressed desire to have children, the postponement of HSCT under intensive surveillance appeared to be the most suiting solution in our case. Since early signs of disease progression cannot currently be detected through classical PB analyses, such as CBC, intensive surveillance often involves relatively invasive procedures like BM aspirate and trephine biopsy, typically performed multiple times a year. Experience with malignant clones in other predisposition syndromes to myeloid malignancies has shown that CBC abnormalities may lag behind the appearance of malignant clones or dysplasia in BM, making BM sampling still essential (30, 31). However, studies are currently ongoing to explore the correlation between BM and PB clonal events (32, 33). The aim is to enable surveillance from PB, which, being less invasive, would allow for more frequent and accurate monitoring of the disease. The patient´s deep understanding even of the complicated aspects of the situation significantly facilitated the decision-making process, especially in a situation where the paucity of data and the lack of evidence-based consensus in the scientific community do not provide a clear management approach.

#### Learning points:

- Pre-emptive HSCT in GATA2 deficiency is associated with excellent outcomes, but still carries risk of potentially life-threatening complications and debilitating diseases.
- Surveillance of patients with GATA2-related MDS still requires regular BM aspirate and biopsy due to the current inability to detect signs of progression from PB alone.
- In situations without a clear HSCT indication, the scarcity of available evidence increases the difficulty in providing patients with a clear treatment algorithm, increasing the importance of shared decision-making.

## CASE 5

A 6-year-old boy presented with early relapse after HSCT of a previously known AML. At first diagnosis the patient had a history of febrile episodes over weeks. Further exams indicated pancytopenia (Hb 7.1 g/dL, WBC 3.1 x 10^9^/L, Plt 39 x 10^9^/L) and 34% blasts in BM. Cytogenetics revealed monosomy 7. The patient was therefore considered high-risk and treated with intensive chemotherapy and allogenic HSCT. The course of the intensive chemotherapy was characterized by episodes of prolonged neutropenia, unclear hyperinflammatory states requiring systemic steroid treatment and several infectious and pulmonary complications, including *Streptococcus mitis* bacteremia with acute respiratory distress syndrome following high-dose cytarabine treatment as well as the development of a consolidation of the left superior pulmonary lobe. Despite an extensive diagnostic work-up including a thoracoscopic wedge-resection no infectious pathogen could be identified. He was transplanted with a BM graft from a 10/10 UD after a conditioning with TTTreoFlu and developed acute GVHD grade III of the skin that resolved after systemic steroid treatment. The routine evaluation at day 100 after HSCT revealed the presence of blasts in PB and BM. At this point, genetic characterization of the leukemic cells detected a mutation in *GATA2* (c.1114G>A [p.A372T]), whose germline origin was subsequently confirmed. The boy received five donor lymphocyte infusions (DLI) in combination with four azacitidine cycles with no significant effect. He was then referred for a second HSCT. The pre-HSCT assessment was primarily aimed at the exclusion of a pulmonary infectious focus and led to the diagnosis of a mucormycosis, with evidence of *Rhizopus species* specifically located in the left inferior lobe. Surgical resection was not feasible and the patient received antifungal treatment with high-dose liposomal amphotericin B and isavuconazole. Despite the uncontrolled infection, a second HSCT was considered as the only curative option, offering the prospect of rapid hematological recovery, fundamental in order to definitely clear the infection. The high risk associated with the procedure was discussed with the family and the patient received PBSCs from a new 10/10 UD after a conditioning regimen with BuCyMel. GVHD prophylaxis was performed with CSA, short-course MTX and serotherapy with ATG. The patient engrafted with complete donor chimerism and a BM analysis demonstrated complete hematological and cytogenetic remission. The first radiologic re-evaluation 45 days after HSCT revealed a slight progress of the mucormycosis. Unfortunately, the patient died 80 days after the second HSCT from a rapidly progressing respiratory failure related to the fungal infection.

### Considerations

This case illustrates the challenges in the diagnosis of rare predisposition syndromes manifesting with advanced myeloid disease in the absence of extra hematological features. In retrospect, the presentation with pancytopenia, rather low blast count and the presence of monosomy 7 should have raised the suspicion of MDS/MDR-AML in the context of a predisposition syndrome.

In the EWOG-MDS(-98) cohort, the use of conventional intensive chemotherapy for patients with MDS-EB before HSCT did not result in improved relapse incidence or non-relapse-related mortality (NRM). The overall survival and event-free-survival were also comparable to those of patients who did not receive intensive chemotherapy before HSCT (7). Furthermore, the use of conventional intensive chemotherapy in patients with GATA2-related MDS is associated with increased NRM probably because of prolonged episodes of neutropenia adding to the risk of the underlying immunodeficiency (4, 23). It is unclear whether the use of single blocks of conventional chemotherapy or novel approaches, such as the use of venetoclax/azacitidine combination therapy with the aim of reducing disease burden prior to HSCT in patients with significantly elevated blast counts (> 20 - 30%) could be beneficial. A recent preclinical study demonstrates increased BCL-2 expression and specific alterations in apoptosis-related proteins in patients with GATA2-related MDS-EB compared to GATA2 patients with RCC or patients with RCC and MDS-EB without GATA2 deficiency. These findings suggest that deregulation of apoptosis may be a potential driver of disease progression in GATA2-related conditions and provide biological evidence for the use of venetoclax-combination therapies in this setting (34). These novel approaches are indeed increasingly adopted and should be objective of future clinical studies (35).

In this patient, the prolonged immunosuppression caused by the AML induction chemotherapy added to the already underlying immunodeficiency predisposing him to the development of serious infectious complications that required surgical intervention and significantly reduced his performance status. The recognition of the underlying predisposition at the time of initial diagnosis would have prompted a less intensive induction treatment and allogeneic HSCT with an intensive myeloablative regimen, possibly contributing to a less complicated course (9).

#### Learning points:

- This case stresses the need to increase the awareness for GATA2 deficiency among colleagues that are routinely involved in the care of hemato-oncologic patients. It is strongly advisable that *GATA2* testing is performed in all children presenting with MDS or AML and monosomy 7, der(1;7) or trisomy 8 in order to minimize the risk of missing the diagnosis (6, 9).
- Conventional intensive chemotherapy in patients with GATA2-related MDR-AML is associated with an increased rate of complications; timely HSCT with intensive myeloablative conditioning, possibly preceded by novel less toxic cytoreductive therapies, should be the preferred approach in these patients.

## Discussion

**Figure 1:**
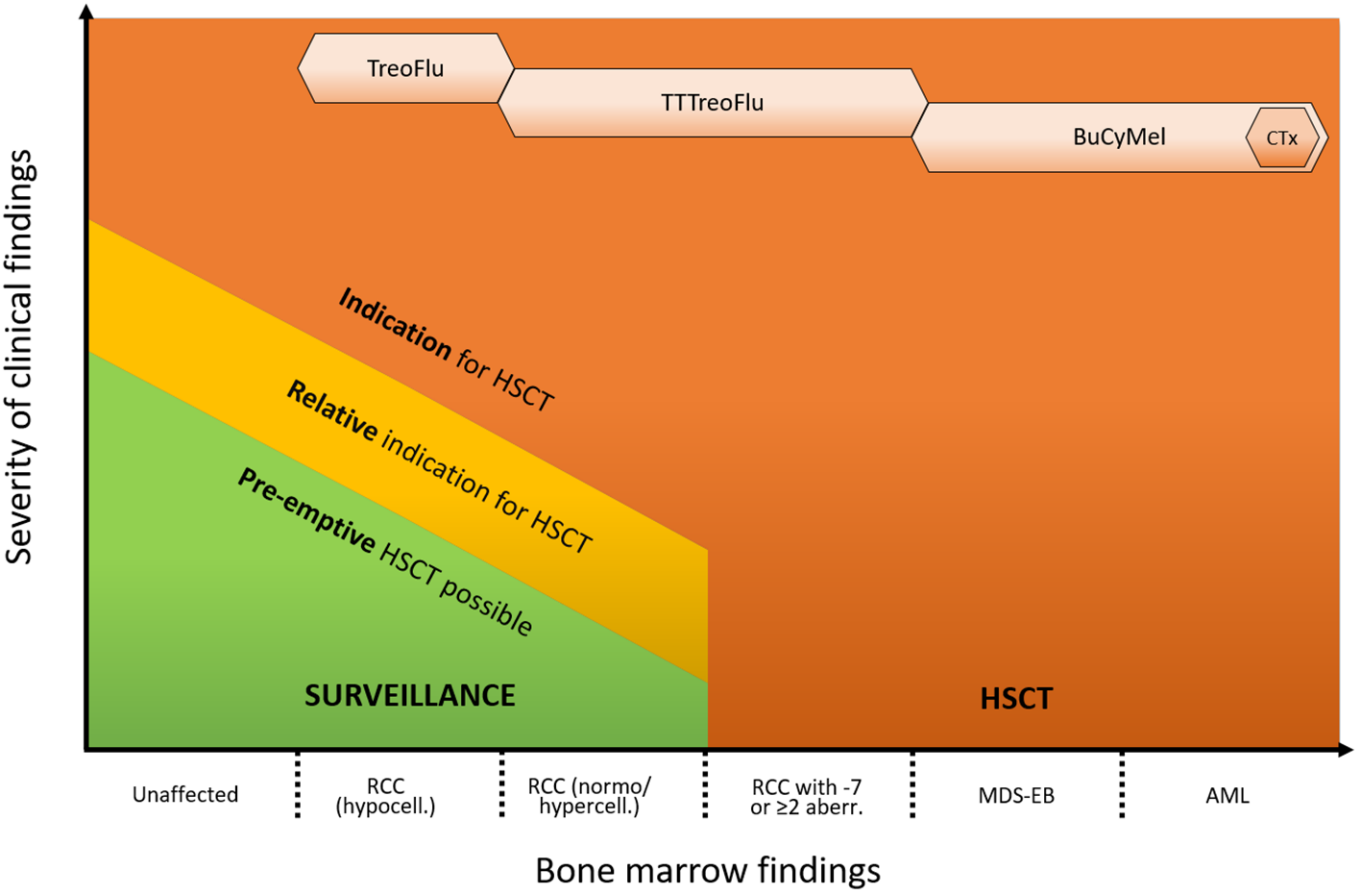
Proposed approach for the management of GATA2 deficiency. The indication for HSCT and the intensity of the conditioning regimen depend on the hematological phenotype and presence of additional clinical symptoms. Indications are advanced hematological disease (MDS-EB/AML, high risk cytogenetics), RCC with transfusion dependency, neutrophil count < 1 x 10^9^/L and/or severe infections. Relative indications are e. g. mild/moderate infections, immune dysregulation or pulmonary alveolar proteinosis. Pre-emptive HSCT can be performed based on individual risk factors (e. g. somatic events/growing clones) or patient’s preference. Monosomy 7 can consist of complete monosomy 7, loss of the long arm of chromosome 7 (del7q) or der(1;7)(q10;p10). HSCT, hematopoietic stem cell transplantation; PAP, pulmonary alveolar proteinosis; RCC, refractory cytopenia of childhood; MDS, myelodysplastic syndrome; AML, acute myeloid leukemia; ANC, absolute neutrophil count; TT, thiotepa; Treo, treosulfan; Flu, fludarabin; Bu, busulfan; Cy, cyclophosphamide; Mel, melphalan; CTx, cytoreductive therapy (e. g. venetoclax and azacitidine).

The cases above illustrate the clinical challenges that have to be faced in the management of patients with GATA2 deficiency as a consequence of its heterogeneous clinical presentation, variable disease course and still incomplete understanding of underlying biology. Possible management strategies for patients with GATA2-related MDS range from watchful waiting to HSCT (**Figure 1**). As suggested by Bortnick et al., the present approach for HSCT in patients with GATA2-related MDS follows the currently recommended EWOG-MDS algorithm for pediatric MDS (6). The authors’ experience indicates that this strategy still remains the most suitable, as it has consistently resulted in very good outcomes over the years. Nevertheless, our small case series emphasizes how the growing recognition of the condition and its increased diagnostic rate have broadened the spectrum of potential variable presentations. Consequently, the number of individuals without a straightforward HSCT indication who still might benefit from the procedure is on the rise. These include for example patients with MDS and immune dysregulation, with (yet) mild or moderate infections, as well as patients with somatic events that are not clearly associated with disease progression.

The open questions in GATA2 deficiency are not limited to the field of MDS. Consensus and evidence is still lacking on the strategy to follow in patients with severe immunodeficiency without MDS, in particular as far as the indication for HSCT and the recommended conditioning regimen are concerned. Similarly, the role of HSCT in treating PAP appears to be growing. The condition seems to improve and in some cases even resolve after HSCT, but larger cohorts are needed to determine the actual efficacy and the optimal HSCT platform in these patients (36). It is reasonable to assume that the threshold for indicating HSCT will change in the coming years, with an increasing proportion of patients choosing pre-emptive HSCT, in particular due to the high risk of progression in GATA2-related MDS. However, despite the most recent advances in the field of transplantation, HSCT remains associated with not negligible morbidity and mortality risks, so that the indication for pre-emptive HSCT in patients with *GATA2* mutation and mild symptoms remains controversial.

To improve patient care and facilitate therapeutic decisions, the scientific community should aim at the development of a more comprehensive knowledge about the multisystem manifestations of GATA2 deficiency, first by collection of international cohorts and establishing registries, in order to better investigate and describe the natural course of the disease and its age-specific problems and risks. Further research should also focus on the understanding of somatic mutations and their adaptive or maladaptive roles in clonal hematopoiesis (37). It is conceivable that not all somatic mutations recurrently found in GATA2 deficiency are associated with progression to advanced disease, with a proportion of them being due to an adaptive but not necessarily malignant mutagenesis. Additionally, there is a need for a better comprehension of the pathophysiology of the disease and the necessity to link the various *GATA2* mutations described so far with their effects on the hematopoiesis, the immune system and the other systems involved, possibly revealing existing genotype/phenotype correlations.

These efforts should finally aim at the development of better risk stratification algorithms in order to allow recognition of early reliable markers of disease progression and support the choice of proper timing for HSCT. The currently available markers, namely the acquisition of high-risk cytogenetic abnormalities and development of blasts in BM, are indeed too late-stage developments and are already linked to inferior treatment outcomes.

In fact, whereas the results of HSCT in patients with RCC and normal karyotype remain excellent, further research is needed to improve the outcomes of the procedure in advanced MDS and in particular MDR-AML. Novel strategies to reduce the disease burden prior to HSCT (e.g. venetoclax and azacitidine), as well as therapies to consolidate the remission after the procedure (e.g. prophylactic azacitidine and DLI) should be explored.

Innovative therapies are currently being studied, including development of medications able to activate compensatory/non-maladaptive signaling pathways and gene editing approaches, with the ultimate aim of prevention rather than cure of the disease (38, 39).

The multisystem involvement of GATA2 deficiency mandates a close cooperation with all involved disciplines, including but not limiting to immunologists and hemato-oncologists, ideally within specialized treating centers. As part of a multidisciplinary management, diagnosis of GATA2 deficiency should always prompt a genetic counseling, in particular for patients or first-grade relatives in childbearing age. Pediatricians in particular should assure that children with GATA2 deficiency receive accurate counseling when reaching adulthood too. Hence, it is crucial to enhance awareness of GATA2 deficiency among medical professionals. This step is vital for delivering improved care and gathering valuable experiences to optimize and personalize treatment strategies for patients grappling with this rare disease.

Our cases underscore the importance of incorporating, whenever feasible, patients and their families into shared decision-making processes. Such an approach should encompass not only the medical aspects, but also consider the psychological and social dimensions of the intricate situation. Understanding the genetic origin of the illness empowers the affected individuals with a coping mechanism against the disease. At the same time, the diagnosis of a genetic predisposition syndrome inevitably heightens the psychological burden and stress on families, for example through the frequent surveillance analyses and the pervasive uncertainty regarding the disease’s progression and its implications for the future of the patient. The possible management options should be thoroughly discussed with the patients and their caregivers and should ideally be re-evaluated with the passing of time, in light of newly emerged needs or most recent understandings of the disease. Finally, it is important to provide patients with tools such as internet platforms that allow for better education, sharing of experiences and advocacy, since optimal care is most effectively achieved through shared decision-making between patients and their caregivers.

## Data availability statement

The original contributions presented in the study are included in the article/supplementary material, further inquiries can be directed to the corresponding author/s.

## Ethics statement

The studies involving humans were approved by Ethik-Kommission der Albert-Ludwigs-Universität Freiburg. The studies were conducted in accordance with the local legislation and institutional requirements. Written informed consent for participation in this study was provided by the participants’ legal guardians/next of kin. Written informed consent was obtained from the individual(s), and minor(s)’ legal guardian/next of kin, for the publication of any potentially identifiable images or data included in this article.

## Author contributions

LV: Conceptualization, Writing – original draft, Writing – review & editing. BS: Formal analysis, Supervision, Writing – review & editing. CS: Validation, Writing – review & editing. ME: Conceptualization, Formal analysis, Funding acquisition, Supervision, Validation, Writing – original draft, Writing – review & editing.

## References

[1] Ripperger T Bielack SS Borkhardt A Brecht IB Burkhardt B Calaminus G. Childhood cancer predisposition syndromes—A concise review and recommendations by the Cancer Predisposition Working Group of the Society for Pediatric Oncology and Hematology. Am J Med Genet Part A. (2017) 173:1017–37. doi: 10.1002/ajmg.a.38142 28168833

[2] Rudelius M Weinberg OK Niemeyer CM Shimamura A Calvo KR. The International Consensus Classification (ICC) of hematologic neoplasms with germline predisposition, pediatric myelodysplastic syndrome, and juvenile myelomonocytic leukemia. Virchows Archiv. (2023) 482:113–30. doi: 10.1007/s00428-022-03447-9 36445482

[3] Arber DA Orazi A Hasserjian RP Borowitz MJ Calvo KR Kvasnicka HM. International Consensus Classification of Myeloid Neoplasms and Acute Leukemias: integrating morphologic, clinical, and genomic data. Blood. (2022) 140:1200–28. doi: 10.1182/blood.2022015850 PMC9479031 35767897

[4] Wlodarski MW Hirabayashi S Pastor V Starý J Hasle H Masetti R. Prevalence, clinical characteristics, and prognosis of GATA2-related myelodysplastic syndromes in children and adolescents. Blood. (2016) 127:1387–97. doi: 10.1182/blood-2015-09-669937 26702063

[5] Spinner MA Sanchez LA Hsu AP Shaw PA Zerbe CS Calvo KR. GATA2 deficiency: a protean disorder of hematopoiesis, lymphatics, and immunity. Blood. (2014) 123:809–21. doi: 10.1182/blood-2013-07-515528 PMC3916876 24227816

[6] Bortnick R Wlodarski M de Haas V De Moerloose B Dworzak M Hasle H. Hematopoietic stem cell transplantation in children and adolescents with GATA2-related myelodysplastic syndrome. Bone Marrow Transplantation. (2021) 56:2732–41. doi: 10.1038/s41409-021-01374-y PMC8563415 34244664

[7] Strahm B Nöllke P Zecca M Korthof ET Bierings M Furlan I. Hematopoietic stem cell transplantation for advanced myelodysplastic syndrome in children: results of the EWOG-MDS 98 study. Leukemia. (2011) 25:455–62. doi: 10.1038/leu.2010.297 21212791

[8] Wachter F Hebert K Pikman Y Yang J Shah B Bledsoe J. Impact of cytoreduction and remission status on hematopoietic cell transplantation outcomes in pediatric myelodysplastic syndrome and related disorders. Pediatr Blood Cancer. (2023) 70:e30530. doi: 10.1002/pbc.30530 PMC13244483 37369986

[9] Locatelli F Strahm B. How I treat myelodysplastic syndromes of childhood. Blood. (2018) 131:1406–14. doi: 10.1182/blood-2017-09-765214 29438960

[10] Collin M Dickinson R Bigley V. Haematopoietic and immune defects associated with GATA2 mutation. Br J Haematol. (2015) 169:173–87. doi: 10.1111/bjh.13317 PMC4409096 25707267

[11] Wehr C Grotius K Casadei S Bleckmann D Bode SFN Frye BC. A novel disease-causing synonymous exonic mutation in GATA2 affecting RNA splicing. Blood. (2018) 132:1211–5. doi: 10.1182/blood-2018-03-837336 PMC6137559 30030275

[12] Kozyra EJ Pastor VB Lefkopoulos S Sahoo SS Busch H Voss RK. Synonymous GATA2 mutations result in selective loss of mutated RNA and are common in patients with GATA2 deficiency. Leukemia. (2020) 34:2673–87. doi: 10.1038/s41375-020-0899-5 PMC7515837 32555368

[13] González-Lara MF Wisniowski-Yáñez A Pérez-Patrigeon S Hsu AP Holland SM Cuellar-Rodríguez JM. Pneumocystis jiroveci pneumonia and GATA2 deficiency: Expanding the spectrum of the disease. J Infection. (2017) 74:425–7. doi: 10.1016/j.jinf.2017.01.005 28126493

[14] Donadieu J Lamant M Fieschi C Fontbrune FSde Caye Aurélie Ouachee M. Natural history of GATA2 deficiency in a survey of 79 French and Belgian patients. haematologica. (2018) 103:1278–87. doi: 10.3324/haematol.2017.181909 PMC6068047 29724903

[15] Nováková M Žaliová Markéta Suková M Wlodarski M Janda Aleš Froňková E. Loss of B cells and their precursors is the most constant feature of GATA-2 deficiency in childhood myelodysplastic syndrome. haematologica. (2016) 101:707–16. doi: 10.3324/haematol.2015.137711 PMC5013954 27013649

[16] Calvo KR Hickstein DD. The spectrum of GATA2 deficiency syndrome. Blood. (2023) 141:1524–32. doi: 10.1182/blood.2022017764 PMC10082373 36455197

[17] Amarnani AA Poladian KR Marciano BE Daub JR Williams SG Livinski AA. A panoply of rheumatological manifestations in patients with GATA2 deficiency. Sci Rep. (2020) 10:8305. doi: 10.1038/s41598-020-64852-1 32433473 PMC7239896

[18] Sallman DA List A. The central role of inflammatory signaling in the pathogenesis of myelodysplastic syndromes. Blood. (2019) 133:1039–48. doi: 10.1182/blood-2018-10-844654 PMC7022316 30670444

[19] Kardos G Baumann I Passmore SJ Locatelli F Hasle H Schultz KR. Refractory anemia in childhood: a retrospective analysis of 67 patients with particular reference to monosomy 7. Blood. (2003) 102:1997–2003. doi: 10.1182/blood-2002-11-3444 12763938

[20] Strahm B Locatelli F Bader P Ehlert K Kremens B Zintl F. Reduced intensity conditioning in unrelated donor transplantation for refractory cytopenia in childhood. Bone Marrow Transplantation. (2007) 40:329–33. doi: 10.1038/sj.bmt.1705730 17589538

[21] EWOG-MDS Consensus Conference. Guidelines for hematopoietic stem cell transplantation (HSCT) in childhood MDS and JMML for patients enrolled in EWOG-MDS studies. In Freiburg;. (2017), 1–19. Available at: https://ewog-mds-saa.org/treatment.html

[22] Galera P Hsu AP Wang W Droll S Chen R Schwartz JR. Donor-derived MDS/AML in families with germline GATA2 mutation. Blood. (2018) 132:1994–8. doi: 10.1182/blood-2018-07-861070 PMC6213320 30232126

[23] Wlodarski MW Collin M Horwitz MS. GATA2 deficiency and related myeloid neoplasms. Semin Hematol. (2017) 54:81–6. doi: 10.1053/j.seminhematol.2017.05.002 PMC5650112 28637621

[24] Fabozzi F Mastronuzzi A Ceglie G Masetti R Leardini D. GATA 2 deficiency: focus on immune system impairment. Front Immunol. (2022) 13:865773. doi: 10.3389/fimmu.2022.865773 35769478 PMC9234111

[25] Ostergaard P Simpson MA Connell FC Steward CG Brice G Woollard WJ. Mutations in GATA2 cause primary lymphedema associated with a predisposition to acute myeloid leukemia (Emberger syndrome). Nat Genet. (2011) 43:929–31. doi: 10.1038/ng.923 21892158

[26] Roncareggi S Girardi K Fioredda F Pedace L Arcuri L Badolato R. A nationwide study of GATA2 deficiency in Italy reveals novel symptoms and genotype–phenotype association. J Clin Immunol. (2023) 43:2192–207. doi: 10.1007/s10875-023-01583-8 37837580

[27] Greenberg PL Tuechler H Schanz J Sanz G Garcia-Manero G Solé F. Revised international prognostic scoring system for myelodysplastic syndromes. Blood. (2012) 120:2454–65. doi: 10.1182/blood-2012-03-420489 PMC4425443 22740453

[28] West RR Calvo KR Embree LJ Wang W Tuschong LM Bauer TR Jr. ASXL1 and STAG2 are common mutations in GATA2 deficiency patients with bone marrow disease and myelodysplastic syndrome. Blood Advances. (2022) 6:793–807. doi: 10.1182/bloodadvances.2021005065 34529785 PMC8945308

[29] Pastor V Hirabayashi S Karow A Wehrle J Kozyra EJ Nienhold R. Mutational landscape in children with myelodysplastic syndromes is distinct from adults: specific somatic drivers and novel germline variants. Leukemia. (2017) 31:759–62. doi: 10.1038/leu.2016.342 27876779

[30] Hakkarainen M Kaaja I Douglas SPM Vulliamy T Dokal I Soulier J. The clinical picture of ERCC6L2 disease: from bone marrow failure to acute leukemia. Blood. (2023) 141:2853–66. doi: 10.1182/blood.2022019425 36952636

[31] Kennedy AL Myers KC Bowman J Gibson CJ Camarda ND Furutani E. Distinct genetic pathways define pre-malignant versus compensatory clonal hematopoiesis in Shwachman-Diamond syndrome. Nat Commun. (2021) 12:1334. doi: 10.1038/s41467-021-21588-4 33637765 PMC7910481

[32] Attardi E Gray N Lewis S Boals M Takemoto CM Sharma R. 2024 ASPHO conference papers and posters. Pediatr Blood Cancer. (2024) 71:e30977. doi: 10.1002/pbc.30977

[33] DeZern AE Goll J Jensen TL Srivatsan SN Gillis NK Abel GA. Correlation between peripheral blood and bone marrow somatic mutations among patients with suspected or established myelodysplastic syndromes from the national MDS study. Blood. (2023) 142:1860–0. doi: 10.1182/blood-2023-178587

[34] Schreiber F Piontek G Schneider-Kimoto Y Schwarz-Furlan S De Vito R Locatelli F. Development of MDS in pediatric patients with GATA2 deficiency: increased histone trimethylation and deregulated apoptosis as potential drivers of transformation. Cancers. (2023) 15(23):5594. doi: 10.3390/cancers15235594 38067298 PMC10705137

[35] Masetti R Baccelli F Leardini D Gottardi F Vendemini F Di Gangi A. Venetoclax-based therapies in pediatric advanced MDS and relapsed/refractory AML: a multicenter retrospective analysis. Blood Adv. (2023) 7(16):4366–70. doi: https://doi.org/10.1182/bloodadvances.2023010113 37216275 10.1182/bloodadvances.2023010113 PMC10432591

[36] van Lier YF de Bree GJ Jonkers RE Roelofs JJTH ten Berge IJM Rutten CE. Allogeneic hematopoietic cell transplantation in the management of GATA2 deficiency and pulmonary alveolar proteinosis. Clin Immunol. (2020) 218:108522. doi: 10.1016/j.clim.2020.108522 32682923

[37] Choijilsuren HB Park Y Jung M. Mechanisms of somatic transformation in inherited bone marrow failure syndromes. Hematology. (2021) 2021:390–8. doi: 10.1182/hematology.2021000271 PMC8791168 34889377

[38] Lundgren S Keränen M Wartiovaara-Kautto U Myllymäki M. Somatic compensation of inherited bone marrow failure. Semin Hematol. (2022) 59:167–73. doi: 10.1053/j.seminhematol.2022.07.002 36115694

[39] Calado RT Clé DV. Treatment of inherited bone marrow failure syndromes beyond transplantation. Hematology. (2017) 2017:96–101. doi: 10.1182/asheducation-2017.1.96 29222242 PMC6142589

